# Evaluation of Serum Interleukin-6 and C-reactive Protein Levels in Drug-naïve Major Depressive Disorder Patients

**DOI:** 10.7759/cureus.3868

**Published:** 2019-01-11

**Authors:** Nuruna Lovely Nishuty, Md. Mehedi Hasan Khandoker, James Regun Karmoker, Sumiya Ferdous, Mohammad Shahriar, M.M.A. Shalahuddin Qusar, Md. Saiful Islam, Mohammad Fahim Kadir, Md Rabiul Islam

**Affiliations:** 1 Department of Pharmacy, University of Asia Pacific, Dhaka, BGD; 2 Department of Psychiatry, Bangabandhu Sheikh Mujib Medical University, Dhaka, BGD; 3 Department of Clinical Pharmacy and Pharmacology, University of Dhaka, Dhaka, BGD

**Keywords:** major depression, inflammatory markers, c-reactive protein, interleukin-6, crp, il-6

## Abstract

Background

Major depressive disorder (MDD) is a disabling health problem with a very high global prevalence and burden. Alteration of inflammatory markers in depression is of growing interest to many psychiatry researchers. This study aimed to examine the serum levels of interleukin-6 (IL-6) and C-reactive protein (CRP) in MDD patients to find out their association with depression.

Materials and methods

The present study recruited 88 MDD patients and 86 control subjects matched by age, gender, and body mass index (BMI). The Hamilton depression rating scale (Ham-D) was used on all patients to measure their severity of depression. Serum levels of IL-6 and CRP were analyzed by commercially available enzyme-linked immunosorbent assay (ELISA) kits (Abcam, Cambridge, MA, USA).

Results

The mean values of serum levels of IL-6 and CRP were 2.94 ± 0.12 pg/mL and 0.99 ± 0.02 mg/L for the patient group and 2.42 ± 0.21 pg/mL and 1.09 ± 0.06 mg/L for the control group, respectively. We found significantly elevated concentrations of serum IL-6 in MDD patients compared with control subjects (p < 0.001). However, the alteration of serum CRP levels was not significant between the groups (p = 0.126). Ham-D scores of patients were positively correlated with serum IL-6 (r = 0.552; p = 0.004) and CRP (r = 0.621; p < 0.001) levels. Moreover, serum IL-6 and CRP levels were observed to be positively correlated (r = 0.452; p = 0.043) with each other in depression.

Conclusions

The present study suggests that increased serum IL-6 level might be a contributing factor to the pathogenesis of depression.

## Introduction

Major depressive disorder (MDD) is a serious mental illness that affects approximately 5% to 20% of the global population [1]. MDD is characterized by a low mood with sadness or irritability accompanied by several psychophysiological changes like disturbances in sleep, appetite, or sexual desire, constipation, loss of the ability to experience pleasure in personal and family life, crying, suicidal thoughts or plans, and slowing of speech and actions [2]. The nature and heterogeneity of the disease confirmed that there is no single etiologic factor involved in the development and progression of depression but rather many factors such as genetics, biochemical, nutritional, and environmental factors [3-5]. Moreover, depression is linked with an increased risk of death from cancer, cardiovascular disease, and diabetes [6]. Therefore, advanced approaches are required for the early diagnosis and appropriate treatment of major depression.

Deregulation of neurochemicals or neuron damage can occur due to the activation of the inflammatory immune responses, and these may contribute to clinical depression [7]. Interleukins enhance neurogenesis under normal physiological condition, but overexposure to these biochemical mediators may cause brain damage, inhibition of neurogenesis, dysfunction of neurotransmitter, and oxidative stress [8]. Inflammation may arise from the over-activated immune system due to chronic stress and depression [9]. Stress-induced inflammation is characteristically different from traditional inflammation, but the same inflammatory markers are released in both cases. In recent years, inflammatory markers have been thought to play a vital role in the development and progression of major depression [10]. Thus, the inflammatory effect on depression has gained major attention over the last few years in the field of psychiatric research.

Evaluation of inflammatory markers in depression has been reported by several studies; increased levels of these markers were observed in most cases. Serum levels of interleukin-6 (IL-6), interleukin-33, C-reactive protein (CRP), tumor necrosis factor-α, and macrophage inflammatory protein-1β were found elevated in people with depression [7]. In addition, one animal model study showed that the direct administration of interleukin-1 beta to the brain is capable of inducing depression [11]. On the other hand, another recent study reported that no associations were observed between depression and serum CRP levels [12] while another study found that plasma IL-6 levels were significantly lower in subjects with depression than non-depressive subjects [13]. Moreover, observing elevated peripheral IL-6 and CRP levels would have promoted the psychoneuroimmunology theory as a contributing factor for the development of depression [14]. Therefore, an association between depression and inflammatory markers is still unclear. Currently, sufficient investigation on the serum levels of IL-6 and CRP as peripheral pro-inflammatory markers has not been conducted. Thus, the present study aimed to measure serum levels of IL-6 and CRP in a relevant Bangladeshi population.

## Materials and methods

Study population

Drug-naïve MDD patients who met the diagnostic criteria according to the Diagnostic and Statistical Manual of mental disorders, fifth edition (DSM-5) were included in this prospective case-control study. Patients were taken from the department of psychiatry, Bangabandhu Sheikh Mujib Medical University (BSMMU), Dhaka, Bangladesh. Healthy volunteers were recruited from the different areas of Dhaka city matched by age, gender, and body mass index (BMI) with patients. A qualified psychiatrist performed the diagnosis of patients and evaluation of control subjects according to DSM-5 using the Structured Clinical Interview for DSM-5. The Hamilton Depression Rating Scale (Ham-D) was used on all patients to measure the severity of their depression via a structured interview [15]. MDD patients with a Ham-D score of seven or more were eligible to enroll in this study. A total of 88 patients and 86 control subjects were included. Additional exclusion criteria for both the patients and controls were chronic physical illness, inflammatory disorders, abnormal BMI, infectious diseases, immune disorders, and substance abuse or dependency.

Blood sample collection and storage

After an overnight fast, a 5-mL blood sample was collected from the cephalic vein of each participant. The samples were then allowed to clot for one hour at room temperature. Serum samples were extracted from the blood sample by centrifugation at 1000 x g for 15 minutes at room temperature. The serum specimens were stored at -80°C until further analysis.

Quantification of serum IL-6 and CRP

Serum IL-6 and CRP concentrations were measured by commercially available enzyme-linked immunosorbent assay (ELISA) kits (Abcam, USA). Preparations of reagents, standard, and serum samples, as well as assay procedure, were performed according to manufacturers’ instruction for the respective ELISA kit. Each plate for an ELISA kit consisted of 96 microtiter wells. All the microtiter wells were coated with antibodies directed towards an antigenic site of a specific cytokine molecule. The concentrations of IL-6 and CRP were expressed as pg/mL and mg/L, respectively. The sensitivity of the assay method is less than 2 pg/mL for both IL-6 and CRP. There was no cross-reactivity with any other cytokines present in the serum sample.

Statistical analysis

The present study used parametric tests (t-test and Pearson’s correlation coefficient) as the descriptive statistics were normally distributed. An independent sample t-test was used for continuous variables and Fisher’s exact test for categorical variables. The Pearson correlation test was used to find the correlation between Ham-D scores and laboratory findings in the patient group. Boxplot graphs were used to compare the study parameters between the cases and controls. Scatter plot graphs were used to show a comparison between serum levels of analyzed markers and Ham-D scores in the patient group. All statistical analyses were two-tailed and conducted using Statistical Package for Social Sciences (SPSS) version 23.0 (IBM Corp., Armonk, NY). The significance level was set at 5%. Results were considered statistically significant if the p-values were less than 0.05.

## Results

Anthropometric and demographic profile

The socio-demographic and biophysical characteristics of the study population are summarized in Table 1. We found that 82% of patients remained in the normal BMI range. Additionally, we observed that 80% of patients had an average monthly family income within 10-25 kilo Bangladeshi taka. Moreover, MDD patients and their corresponding controls were similar in terms of their age, gender, BMI, level of education, occupation, income, and smoking habits.

**Table 1:** Anthropometric and demographic profile of the study population Significant p-values ≤ 0.05 at 95% confidence interval. BMI: Body mass index; CED: Chronic energy deficiency; KBDT: Kilo Bangladeshi taka; SEM: Standard error of the mean; N: Number.

Parameters	Patients (N = 88)				Controls (N = 86)			p-value
	N	%	Mean ± SEM		N	%	Mean ± SEM	
Age in years			33.32 ± 1.24				31.00 ± 1.44	0.300
18-24	26	29			24	28		
25-34	29	33			25	28		
35-44	23	26			29	34		
45-60	10	12			8	10		
Gender								0.625
Female	46	52			48	56		
Male	42	48			38	44		
BMI (kg/m^2^)			23.00 ± 0.49				23.99 ± 0.90	0.312
Below 18.5 (CED)	8	9			11	13		
18.5-25 (normal)	72	82			62	72		
Above 25 (obese)	8	9			13	15		
Education								0.101
Illiterate	2	2			2	2		
Can read only	11	13			13	15		
Secondary	28	32			25	29		
Higher secondary	22	25			18	21		
Graduate or above	25	28			28	33		
Occupation								0.577
Service	13	15			9	11		
Business	5	6			8	9		
Student	10	11			14	16		
Others	14	16			21	24		
Jobless	46	52			34	40		
Monthly income in KBDT			25.87 ± 3.37				24.91 ± 3.28	0.871
Below 10	8	9			10	12		
10-25	70	80			59	68		
26-40	7	8			11	13		
Above 40	3	3			6	7		
Smoking habit								0.111
Nonsmoker	68	77			61	71		
Smoker	20	23			25	29		

Serum levels of inflammatory markers and clinical outcomes

**Figure 1:**
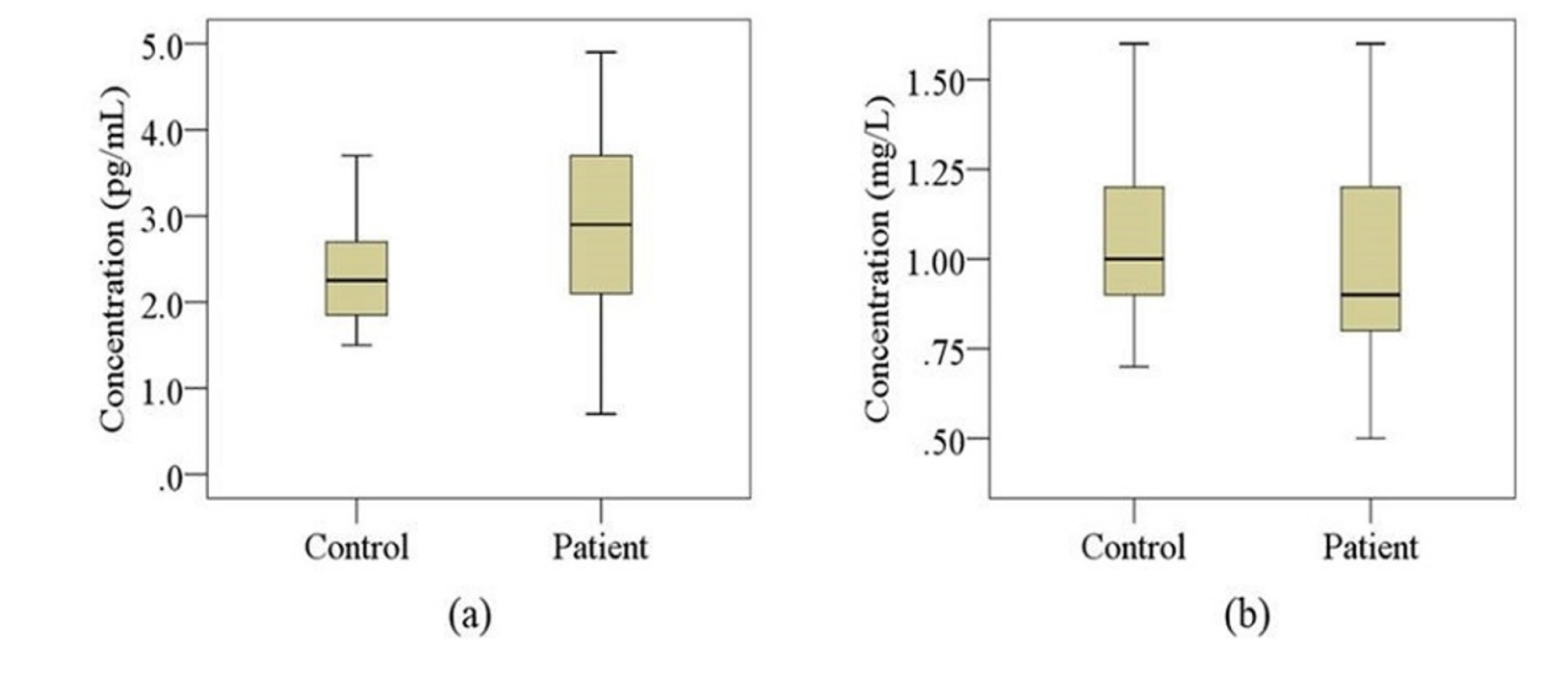
Changes in serum levels of interleukin-6 (IL-6) and C-reactive protein (CRP) in the study population Boxplot showing the median, maximum, and minimum value range. (a) IL-6, (b) CRP. IL-6: Interleukin-6; CRP: C-reactive protein.

Laboratory findings and clinical outcomes are presented in Table 2. Serum levels of IL-6 were found to be significantly increased in MDD patients compared with control subjects (p < 0.001), but no significant difference was observed for serum CRP levels between the groups. Boxplot graphs were used to show the changes in analyzed markers in MDD patients and control subjects where median, maximum, and minimum value ranges are indicated (Figure 1).

**Table 2:** Laboratory and clinical outcomes in major depressive disorder (MDD) patients and control subjects Significant p-values ≤ 0.05 at 95% confidence interval; values in bold: p < 0.05. MDD: Major depressive disorder; SEM: Standard error of the mean; IL-6: Interleukin-6; CRP: C-reactive protein; Ham-D: Hamilton Depression Rating Scale; N: Number.

Parameter	Patient group (N = 88)	Control group (N = 86)	
	Mean ± SEM	Mean ± SEM	p-value
Serum IL-6 (pg/mL)	2.94 ± 0.12	2.42 ± 0.21	< 0.001
Serum CRP (mg/L)	0.99 ± 0.02	1.09 ± 0.06	0.126
Ham-D score	17.12 ± 0.53	4.70 ± 0.24	< 0.001

Relation among different research parameters in the study population

**Figure 2:**
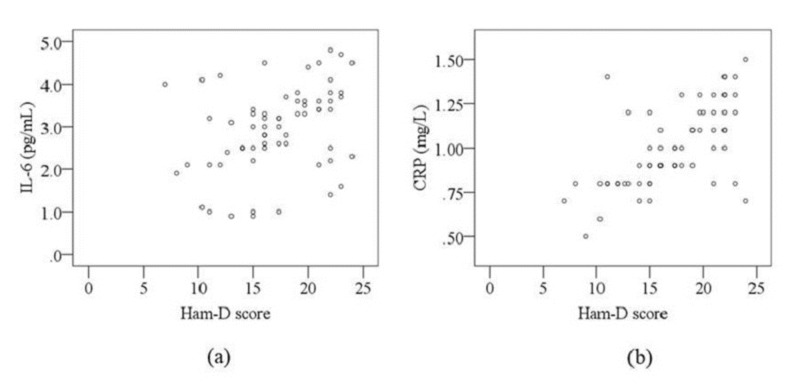
Scatterplot of serum levels of interleukin-6 (IL-6) and C-reactive protein (CRP) in relation to Hamilton Depression Rating Scale (Ham-D) score in major depressive disorder (MDD) patients (a) Correlation between IL-6 and Ham-D score (r = 0.552; p = 0.004). (b) Correlation between CRP and Ham-D score (r = 0.621; p < 0.001). Ham-D: Hamilton Depression Rating Scale; IL-6: Interleukin-6; CRP: C-reactive protein; MDD: Major depressive disorder.

The Pearson correlation analysis showed that Ham-D scores were positively correlated with serum levels of IL-6 (r = 0.552; p = 0.004) and CRP (r = 0.621; p < 0.001) in the patient group (Table 3). Furthermore, serum levels of IL-6 and CRP were positively correlated with each other in MDD patients (r = 0.452; p = 0.043). No significant association was observed among different study parameters in the control subjects. Graphical illustrations of this correlation between Ham-D scores and serum IL-6 levels and between Ham-D scores and serum CRP levels are presented in Figure 2.

**Table 3:** Correlation study among various research parameters in patient and control subjects r = Correlation co-efficient; significant p-values ≤ 0.05 at 95% confidence interval; values in bold: p < 0.05. Ham-D: Hamilton Depression Rating Scale; IL-6: Interleukin-6; CRP: C-reactive protein; N: Number.

Correlation parameters	Patients group (N = 88)		Control group (N = 86)	
	r	p-value	r	p-value
Ham-D score and IL-6 level	0.552	0.004	0.385	0.063
Ham-D score and CRP level	0.621	< 0.001	0.154	0.471
IL-6 and CRP level	0.452	0.043	0.257	0.252

## Discussion

According to the current study results, the mean value of serum IL-6 levels was found to be significantly increased in the patient group compared with control subjects (p < 0.05). This difference can be explained by several psycho-neuro-inflammatory mechanisms. In depression, the hypothalamic-pituitary-adrenal axis is activated by the inflammatory response system (IRS). Thus, corticotropin-releasing hormone and adrenocorticotropic hormone are produced, increasing the turnover of catecholamine and serotonin [16].

On the other hand, in response to immune activation, pro-inflammatory cytokines are produced by T-cells, natural killer cells, and macrophages to regulate the IRS [17]. Multiple studies have shown the increased levels of inflammatory cytokines in depression. For instance, elevated levels of IL-6 and IL-6 receptor antagonist were observed in treatment-resistant MDD patients [18-19]. The present study findings, as well as the study results of Maes et al., are supported by some recent case-control studies. Increased serum concentrations of IL-6 were found in patients suffering from major depression [20-22]. The present study observed a significant positive correlation between study parameters and the severity of depression, also supported by many previous study results. Serum concentration of IL-6 was found to be positively correlated with Ham-D scores or the severity of depression [20]. Some other studies also found a positive correlation between the severity of depression and the serum concentrations of IL-6 [23-24].

The present study observed no significant changes in serum CRP levels between patients and control subjects. This finding is consistent with several previous studies. One such study evaluated serum CRP levels in MDD patients and control subjects but did not find any significant change [12]. Another cross-sectional study conducted by Takekawa et al. found lower levels of serum CRP in depression than in healthy controls, but the deviation was not statistically significant [13]. Similarly, another study examined the association between the severity of depression and serum CRP levels. They also found a significant positive correlation between serum CRP levels and the severity of depression [25].

Furthermore, we recruited drug-naïve MDD patients as serum levels of IL-6 and CRP can be influenced by antidepressant medications. Also, baseline CRP levels are affected by antidepressant therapy in MDD patients [26]. Another study found that serum IL-6 level decreased via the effects of paroxetine in patients with psychological symptoms [27]. Dietary supplementation and lifestyle can be considered as key factors for depression, but we did not examine the effect of these factors on our study parameters. This might be considered as a drawback of the present study. The lack of pre- and post-treatment evaluation of IL-6 and CRP can also be considered as a weakness.

Our study findings provided additional evidence that significantly altered IL-6 activity may be involved to the pathophysiology of depressive disorder. However, much more work in this area still needs to be done to explore the actual association of analyzed parameters with the pathophysiological mechanism of depression.

## Conclusions

To the best of our knowledge, this is the first-ever study on Bangladeshi patients examining the association of serum IL-6 and CRP levels with depression. A significant elevation of serum IL-6 levels was observed in patients with major depression, but the change in serum CRP levels was not significantly different between the groups. These alterations in serum inflammatory cytokine levels might be involved in the pathogenesis of depression. Based on our study results, we believe that significantly elevated serum concentrations of IL-6 are associated with depression. As this is a preliminary study, further studies with a larger and more homogeneous sample are required to establish this finding.
